# Living and Dead Microorganisms in Mediating Soil Carbon Stocks Under Long-Term Fertilization in a Rice-Wheat Rotation

**DOI:** 10.3389/fmicb.2022.854216

**Published:** 2022-06-10

**Authors:** Jie Chen, Dali Song, Haoan Luan, Donghai Liu, Xiubin Wang, Jingwen Sun, Wei Zhou, Guoqing Liang

**Affiliations:** ^1^Ministry of Agriculture Key Laboratory of Plant Nutrition and Fertilizer, Institute of Agricultural Resources and Regional Planning, Chinese Academy of Agricultural Sciences, Beijing, China; ^2^College of Forestry, Hebei Agricultural University, Baoding, China; ^3^Institute of Plant Protection and Soil Fertility, Hubei Academy of Agricultural Sciences, Wuhan, China

**Keywords:** microbial community, long-term fertilization, soil amino sugars, network analysis, partial least squares path model

## Abstract

Although soil microorganism is an active area of research, we are still in the early stages of understanding how living microorganisms influence the accumulations of soil microbial residues under different agricultural practices. Based on a 39-year fertilization experiment, we characterized the soil microbiota and correlated their compositions to soil microbial residues, which are indicated by amino sugars under a rice-wheat rotation. In the present study, fertilization regimes and crop season all exerted significant impacts on the compositions of soil microbial communities and their residues, although no significant difference in the microbial residues was found between soil depth (0–10 cm vs. 10–20 cm). Compared within fertilization regimes, the long-term fertilization, especially the application of organic manure, stimulated the accumulations of carbon (C) and nitrogen in soils and microbial residues. Upland soils in wheat season accumulated more microbial residues, particularly in fungal residues, than paddy soils in rice season. Our results suggested that the long-term application of organic manure favored the growth of soil microbial communities, and then increased the contents of microbial residues, particularly in fungal residues, leading to an enlargement of soil C pools. The keystone taxa *Pseudaleuria* identified by network analysis showed a significantly positive potential in soil C sequestration by increasing the accumulation of fungal residues. Thus, this study revealed the strong and close connections between microbial communities and their residues, and provided evidence about the critical role of keystone taxa in regulating C sequestration.

## Introduction

Soil organic carbon (SOC), retained in the largest pool of terrestrial carbon, is critical in improving soil fertility, maintaining ecosystems sustainability, and mitigating climate change (Schimel and Schaeffer, 2012; Keenan and Williams, 2018). Recent developments have raised the attention in exploring the processes of SOC transformation and sequestration (Miltner et al., 2012; Liang et al., 2019), which can be influenced by various factors, e.g., environmental conditions, edaphic factors, microorganism, and human interference (Schimel and Schaeffer, 2012; Wiesmeier et al., 2019). Empirical studies have suggested the important role of microorganisms in SOC sequestration through microbial metabolism and their residues (Naylor et al., 2020). For instance, living soil microbes usually participate in SOC sequestration by converting plant detritus, root exudates, and other nutrients into microbial cellular components and byproducts, while dead microbes (microbial residues) generally have organic-mineral bonds that stabilize their C content (Liang et al., 2017). Current estimates suggested that soil microbial residues contribute approximately 30–60% to SOC, fungal residues C (FRC) contribute approximately 20–50%, and bacterial residues C (BRC) contribute approximately 10–30% (Liang et al., 2019). Hence, it is necessary to clarify the changes in microbial residues to further evaluate the soil C stocks.

A number of studies have focused on the mechanism of microbial residues affected by abiotic factors, e.g., temperature (Chen G. P. et al., 2020), precipitation (Fierer, 2017), soil pH (Hu et al., 2018), nutrient availability (He et al., 2011), and redox status (Xia et al., 2019), but the biotic factors in regulating the accumulations of microbial residues are still uncertain. Previous studies have reported that the microbial cell walls with a greater proportion of recalcitrant components, such as peptidoglycan in gram-positive bacteria and chitin in fungal cell walls, were considered to be more resistant to decomposition (Kögel-Knabner, 2002). Bacteria and fungi, as the widely spread microbiota, shared a microhabitat and shaped the biological communities above and below the Earth’s surface (Nazir et al., 2009; Frey-Klett et al., 2011), leading to a further change in organic C accumulation and stability. Although there is no strong difference between the turnover of bacterial and fungal residues (Joergensen, 2018), the interactions between living microorganism and their residues remain unclear and elusive, which needs further evidence.

The agroecosystem, which contained 16.32 ± 0.41 Pg C in croplands and accounted for 20.6% of the total terrestrial C pool in China (Tang et al., 2018), could regulate the global C balance through different agricultural practices, e.g., crop rotation (Schimel and Schaeffer, 2012), fertilization regimes (Ye et al., 2019), as well as soil depth (Roth et al., 2011). Soils in crop rotation, especially under the rice-wheat rotation, are typified by high heterogeneity in the distribution of soil nutrients and ecological niches, which can promote the changes in the microbial community biomass and compositions, as well as microbial functions in C cycling (Pan et al., 2003; Schimel and Schaeffer, 2012). A large-scale research on the distribution of microbial residues in paddy and upland soils throughout eastern China suggested that paddy soils were enriched with a greater proportion of bacterial residues, whereas upland soils were replenished by fungal residues (Chen et al., 2021). The long-term inorganic fertilization usually decreased the relative abundances of functional genes that participated in soil C and N cycling, while organic manure increased their abundances (Jiao et al., 2019) and the accumulations of microbial residues (Ye et al., 2019). Current studies also suggested a decrease in soil C accumulations in microbial residues with the increase in soil depth (Peixoto et al., 2020; Gies et al., 2021). Thus, based on the critical role of living and dead microorganisms in soil C cycling, understanding how agricultural practices impact the interactions between living microbiota and microbial residues is an important subject toward a more sustainable agroecosystem.

Amino sugars that can be hydrolyzed from microbial cell walls have been extensively used as proxies for soil microbial residues (Liang et al., 2019). Muramic acid (MurN) occurs exclusively in peptidoglycans in bacterial cell walls (Vollmer et al., 2008), glucosamine (GlcN) primarily originates from fungal chitin (Parsons, 1981), while galactosamine (GluN) originates from both bacterial and fungal cell wall (Engelking et al., 2007). Hence, we quantified the bacterial and fungal residues in soils based on the contents of MurN and GlcN, respectively (Appuhn and Joergensen, 2006; Joergensen, 2018). Moreover, the Python language package Networkx provided a useful tool for exploring and analyzing the complicated connections between soil variables and microbial communities.^1^ Therefore, we carried out this study on the compositions of bacterial and fungal communities and the content of C and N in the soil and microbial residues based on a 39-year fertilization experiment with a rice-wheat crop rotation. This study aimed to (1) explore how the long-term fertilization regimes, crop season, and soil depth affected the compositions of living and dead microorganisms and (2) evaluate the influence of fertilization regimes, crop season, and soil depth on soil C pools by mediating soil living and dead microorganisms. Considering the different fertilization regimes and heterogeneous microenvironment in the rice-wheat rotation, we hypothesized that (i) fertilization regimes, crop season, and soil depth exerted impacts on the soil microbial community compositions, and then altered the contents and components of microbial residues and (ii) keystone taxa influencing soil microbial residues also affected the size of soil C pools.

## Materials and Methods

### Study Site and Soil Sampling

A long-term field experiment, i.e., a rice-wheat cropping system, was established at Ezhou (114.7°E, 30.4°N), Hubei Province, China, in 1981. Wheat is sown in late November and harvested in late May, and rice is sown in early June and harvested in early October. According to the National Data Center for Meteorological Sciences,^2^ from 2019 to 2020, the average precipitation was 3.2 and 2.7 mm, and the average temperature was 27.3 and 12.5^°^C in rice and wheat season, respectively, per day. The soil is derived from yellow-brown paddy soil, classified as Cambisols (Nachtergaele, 2017). The soil before cultivation in 1981 had the following properties: pH 6.3, SOC 15.9 g kg^–1^, TN 1.8 g kg^–1^, available phosphate (AP) 5.0 mg kg^–1^, available potassium (AK), 98.5 mg kg^–1^. The experiment had a complete randomized design with different fertilization regimes and three replicates. Each plot measured 5 m × 8 m. The fertilization regimes in this study contained: (1) no fertilizer, NoF, (2) inorganic nitrogen, phosphate and potassium fertilizers, NPK, (3) manure, M, and (4) the application of NPK combined with manure, NPKM. We applied urea, superphosphate, and potassium chloride as N, P, and K fertilizers, respectively. The detailed application rates of fertilizers are displayed in Table 1. The organic manure was mainly composed of swine manure (69% moisture) and had the following properties: 262.2 g kg^–1^ SOC, 15.1 g kg^–1^ TN, 20.8 g kg^–1^ phosphorous pentoxide, and 13.6 g kg^–1^ potassium dioxide.

**TABLE 1:** Application rates of fertilizers in the long-term experiment.

	Rice season (kg ha^–1^)						Wheat season (kg ha^–1^)					
Fertilization time	Before cultivation				Tillering stage	Elongation stage	Before cultivation				Tillering stage	Elongation stage
	N	P_2_O_5_	K_2_O	Manure	N	N	N	P_2_O_5_	K_2_O	Manure	N	N
NoF	–	–	–	–	–	–	–	–	–	–	–	–
NPK	36	45	90	–	36	18	30	30	60	–	15	15
M	–	–	–	11,250	–	–	–	–	–	11,250	–	–
NPKM	36	45	90	11,250	36	18	30	30	60	11,250	15	15

### Soil Sampling and Analysis

Five intact soil cores were collected in October 2019 and May 2020, using stainless steel cores (5 cm inner diameter × 10 cm height) from a depth of 0–10 and 10–20 cm in each plot, which finally contained 48 samples (4 fertilization regimes × 2 crop season × 2 soil depth × 3 replicates). The fresh soil was transported to the laboratory immediately, sieved through a 2 mm mesh, and divided into three subsamples. One subsample was prepared for microbial DNA extraction, one was stored at 4^°^C for the analysis of extracellular enzyme activity, and the third was air-dried to analyze the soil physicochemical properties. SOC and TN were determined by an Elemental Analyzer (VARIO MACRO cube, Germany).

### Soil Amino Sugar Analysis

The extraction of soil amino sugars, including GlcN, GalN, and MurN, was determined following Zhang and Amelung (1996). Briefly, a weighted air-dried soil sample (contained about 0.3 mg N) was hydrolyzed with 6 mol l^–1^ HCl at 105^°^C for 8 h, and then cooled to room temperature. The solution was mixed with 100 μl inositol and dried by a rotatory evaporator. The precipitate was dissolved in distilled water, adjusted to pH 6–6.8, centrifuged at 3,000 r min^–1^, and freeze-dried. Then, methanol was added to freeze-dried supernatants, centrifuged at 3,000 r min^–1^, transferred to vials, and dried by N_2_ at 45^°^C. Next, the residue was dissolved in 1 ml distilled water and 100 μl N-methylglucamine, mixed, and freeze-dried. The purified amino sugars were reacted with ethyl acetate and n-hexane. The amino sugar derivatives were separated on a gas chromatograph equipped with an HP-5 column (30 m × 0.25 mm × 0.25 mm) and quantified using a flame ionization detector (GC-7890B, Agilent, United States). The temperature program was set as follows: 120^°^C for 1 min, increased by 10^°^C min^–1^ to 250^°^C and held for 2.5 min, increased by 20^°^C min^–1^ to 270^°^C and held for 2 min. The injector temperature was 250^°^C and the split ratio was 10: 1. The peaks were identified by comparing sample retention times with those of pure amino sugar standards using the Chemstation software. Myo-inositol was added as the first standard after hydrolysis and N-methylglucamine was added as the second standard before the derivatization to assess the recovery of amino sugars.

The contents of amino sugars, microbial residue-C and N were calculated using the analytical equations developed by Appuhn and Joergensen (2006) and Joergensen (2018).


mx=miAx/AiRf

BRC=MurN×45

Bacterial⁢residues-N=MurN×6.67

$$\text{FRC}=\left(\frac{\text{GlcN}}{179.17}-2\times \frac{\text{MurN}}{251.23}\right)\times 179.17\times 9$$

$$\mathrm{Fungal}\,\mathrm{residues}-N=\left(\frac{\text{GlcN}}{179.17}-2\times \frac{\text{MurN}}{251.23}\right)\times 179.17\times 1.4$$

where m_*i*_ represents the quality of inositol, and A_*i*_ and A_*x*_ present the peak area of inositol and amino sugars, respectively. In addition, 45 is the conversion coefficient of MurN to BRC, 6.67 is the conversion coefficient of MurN to bacterial residues-N (BRN), 179.17 is the molecular weight of GlcN, 9 is the conversion coefficient of fungal GlcN to FRC, and 1.4 is the conversion coefficient of GlcN to fungal residues-N (FRN).

### Amplicon Sequencing and Bioinformatics

DNA was extracted from 500 mg soil using the FASTDNA Spin Kit (MP Biomedical, Santa Ana, CA, United States) according to the manufacturer’s protocols. The DNA concentration and quality were checked by NanoDrop (Thermo NanoDrop, 2000, United States). For bacteria, the V3-V4 hypervariable regions of the 16S rRNA gene were targeted using primers 515F (GTGCCAGCMGCCGCGGTAA) and 806R (GGACTACVSGGGTATCTAAT); for fungi, the ITS1 region was amplified using ITS1F (CTTGGTCATTTAGAGGAAGTAA) and ITS2R (GCTGCGTTCTTCATCGATGC). Sequencing was performed on the Illumina MiSeq platform. Sequenced paired-end reads were joined using FLASH^3^ and were filtered according to quality using Fastp tools.^4^ Briefly, sequences were joined (overlapping pairs) and grouped by samples following the barcodes and then the barcodes were removed. Then, sequences <50 bp or with ambiguous base calls were removed. Reads were denoised by unoise3, and any chimeric sequences were removed using the USEARCH tool based on silva_16s_v123. Sequences were then split into operational taxonomic units (OTUs) at 97% similarity using the UPARSE pipeline in USEARCH. Plastid and non-bacteria were removed by USEARCH based on silva_16s_v123. This involved quality filtering and 97% clustering of the ITS1 region as indicated above for the 16S processing, using the UNITE database (utax_reference_dataset_all_02.02.2019), for chimera removal, taxonomic identification, and non-fungal removal of representative OTUs. The bacterial and fungal OTU abundance tables were rarefied to an even depth of 30,000 reads for 16S and 25,000 for ITS sequences. The raw sequencing data were deposited in the NCBI Sequence Read Archive (SRA)^5^ with accession No. PRJNA796782.

### Statistical Analysis

The data were processed using Microsoft Excel 2019 and the analysis of variance (ANOVA) was conducted by using SPSS 18.0 (IBM, United States), followed by a Fisher’s least significant difference (LSD) test at *p* < 0.05 to determine the effects of fertilization regimes, crop season, and soil depth on the contents of C and N in soil and microbial residues. PERMANOVA was conducted using the *adonis* () function with 9,999 permutations, variance portioning analysis (VPA) was determined using the *varpart* () function, and differences within-group centroids were calculated using the *betadisper* () function. All these functions were from the ‘‘vegan’’ package^6^ in R (version 3.6.1). Principal co-ordinates analysis (PCoA) was conducted to calculate the differences within microbial community compositions using the *BetaDiv* () function in “phyloseq” package, which was based on the Bray-Curits distance (Adonis test) with 999 permutations.

Partial least squares path model (PLS-PM) was performed using the “plspm” package (Sanchez et al., 2015) to further investigate the potential direct and indirect effects of inorganic and organic fertilization, crop season, and soil depth on soil C and N contents by influencing the compositions of microbial communities and their residues, which finally influence soil C pools. Bacterial community was represented by the sample values of PC1 and PC2 derived from PCoA analysis. Bacterial residues contained BRC and BRN, while fungal residues contained FRC and FRN. The quality of the PLS-PM was evaluated by examining the goodness of fit (GoF) index, in which >0.7 means an acceptable overall prediction performance of the model, and by calculating the coefficients of determination of the variables (R^2^). Then, we estimated the standardized total effects of all variables upon soil SOC and TN by using the “pls” package.

To explore the complex relationships between microbial communities and their residues, we further constructed the networks using the Python language package Networkx (see text footnote 1). Only OTUs with top 500 abundances were considered. Edges were represented as links between microbial OTUs and the soil variables, which were tested by the Pearson correlation analysis. Only edges with *p* < 0.05 and |ρ| > 0.5 were included in the final networks. The network properties, including node degree, degree centrality, closeness centrality, and betweenness centrality, were also calculated. A node with the highest score of closeness centrality, betweenness centrality, and degree centrality are indicated as keystone taxa (Chen et al., 2020). And the networks were visualized using the Matplotlib Python package.^7^

Some figures were plotted using OriginPro 9 (OriginLab, United States) and modified using Adobe Illustrator CS6 (Adobe, United States).

## Results

### Changes in the Contents of Soil C and N

Fertilization regimes and crop season exerted significant impacts on the contents of SOC and TN (*p* < 0.05), while soil depth did not exert any impact (Table 2, *p* > 0.05). For fertilization regimes, compared with the original soil, the long-term fertilization enlarged SOC and TN pools, with an increase of 3.8–31.4% and 1.5–19.2%, respectively, while the long-term non-fertilization significantly decreased, with an average decrease of 15.8 and 6.6%, respectively. Compared with soils in NPK, the long-term application of organic manure, especially NPKM, significantly increased SOC and TN contents by 42.2 and 24.6%, respectively. Compared with soils in rice season, a relatively higher TN content was observed in wheat fields, which increased by 12.7%.

**TABLE 2:** The average contents and ratios of C and N in soils and microbial residues.

Treatments			SOC (g kg^–1^)	TN (g kg^–1^)	BRC (g kg^–1^)	BRN (g kg^–1^)	FRC (g kg^–1^)	FRN (g kg^–1^)	BRC/SOC	BRN/TN	FRC/SOC	FRN/TN
Original soil			14.2	1.84	0.92	0.14	5.15	0.8	6.5	7.4	36.3	43.5
Rice season	0–10 cm	NoF	12.3	1.85	1.64	0.25	4.76	0.74	13.4	13.8	38.7	41.5
		NPK	15.9	1.95	2.14	0.32	4.99	0.77	13.5	16.3	31.5	39.7
		M	15.9	2.01	2.06	0.3	5.9	0.92	13.2	15.2	37.6	45.8
		NPKM	19.7	2.41	2.13	0.32	6.31	0.98	10.9	13.3	32.1	40.6
	10–20 cm	NoF	11.6	1.62	1.36	0.2	3.39	0.53	11.8	12.4	29.5	32.5
		NPK	13.1	1.63	1.19	0.18	3.1	0.48	9.1	10.9	23.7	30.0
		M	12.6	1.69	1.68	0.25	4.03	0.63	13.6	14.8	32.2	37.6
		NPKM	21.8	1.76	2.07	0.31	4.84	0.75	9.5	17.6	22.3	42.5
Wheat season	0–10 cm	NoF	10.9	1.64	1.92	0.29	5.52	0.86	18.3	17.9	51.8	53.1
		NPK	16.0	2.04	1.93	0.29	5.42	0.84	12.1	14.4	33.8	41.6
		M	16.3	2	2.15	0.32	5.69	0.89	13.2	16.0	34.8	44.3
		NPKM	19.3	2.35	2.16	0.32	6.28	0.98	11.3	13.9	32.7	41.9
	10–20 cm	NoF	12.9	1.78	1.97	0.29	4.95	0.77	15.6	16.7	38.6	43.3
		NPK	13.8	1.87	2.16	0.32	6.53	1.02	15.7	17.1	47.4	55.0
		M	23.4	2.83	2.42	0.36	7.37	1.15	10.4	12.8	31.5	40.4
		NPKM	21.9	2.6	2.96	0.44	7.89	1.23	13.5	17.0	36.0	47.2
*p*-value	Crop rotation		ns	**	***	***	***	***	*	ns	***	***
	Soil depth		ns	ns	ns	ns	ns	ns	*	ns	***	***
	Fertilization regimes		***	**	**	**	**	**	ns	ns	ns	ns
NoF			c	c	b	b	b	b				
NPK			b	bc	b	b	ab	ab				
M			b	ab	ab	ab	ab	ab				
NPKM			a	a	a	a	a	a				

Significant effects were tested by LSD t-test. Asterisk indicates significance, * p < 0.05, ** p < 0.01, *** p < 0.001.

For the contents of soil microbial residues, fertilization regimes and crop season provided significant contributions to their variations, especially crop rotation. Meanwhile, soil depth only exerted significant impacts on the contents of FRC and FRN. In detail, when compared with original soil, the long-term fertilization significantly increased the contents of BRC and BRN by 29.0–220.5% and 28.9–221.0%, respectively, while NPKM significantly increased the FRC and FRN by an average of 13.4 and 13.5%, respectively. Compared with NoF, NPKM significantly increased the accumulations of BRC, BRN, FRC, and FRN by 12.7–52.6%, 12.8–53.3%, 13.7–59.2%, and 13.6–59.3%, respectively. For crop season, the accumulations of microbial residues were relatively higher in the wheat fields, and the contents of BRC, BRN, FRC, and FRN were on average 24.2, 24.4, 33.7, and 33.8% higher than those in the rice fields. Compared with the upper soil layer, the contents of FRC and FRN were significantly higher in the lower layer with an increase of 33.7 and 33.8%, respectively.

Moreover, the ratios of microbial residues to SOC and TN can reflect the contributions of the bacterial or fungal residues to soil C and N pools. The ratios of FRC/SOC and FRN/TN accounted for 23.7–51.1% and 30.0–55.0%, respectively, which were significantly higher than the ratios of BRC/SOC (9.1–15.7%) and BRN/TN (10.9–19.9%).

### Changes in the Living Soil Microbial Communities

**FIGURE 1:**
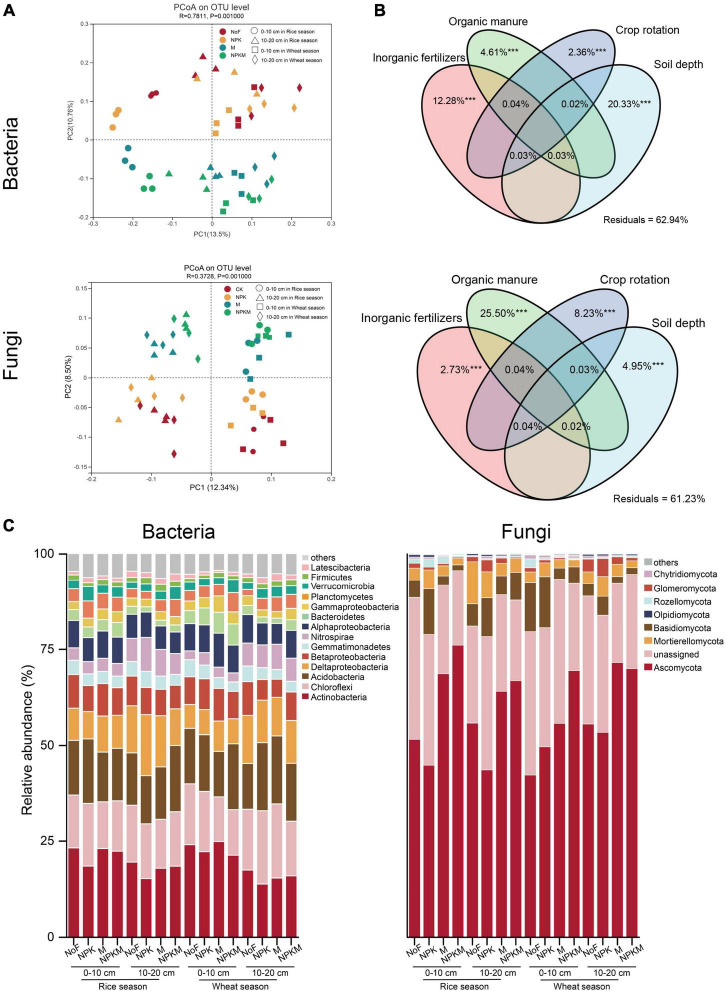
Soil bacterial and fungal community compositions. **(A)** PCoA analysis of soil bacterial (upper) and fungal (lower) community compositions. **(B)** Variance partitioning analysis (VPA) of the bacterial (upper) and fungal (lower) communities by inorganic fertilization, organic manure, crop rotation, and soil depth. Values < 0 were not shown. *** indicated significance at *p* < 0.001. **(C)** Taxonomic profiles of bacterial and fungal communities at phylum level. Bacteria and fungi phyla with relative abundances lower than 1% were summarized with “others” (Bray-Curtis).

Differences in the overall microbial community compositions based on the PCoA revealed that both bacterial and fungal community compositions demonstrated a significant structuring in response to fertilization regimes, crop season, and soil depth (Figure 1A and Table 3). In detail, soil depth made the highest contribution to the variation of bacterial community compositions, and organic manure contributed more to the variation of fungi (Figure 1B). Major bacterial and fungal phyla were observed in all treatments (Figure 1C). Within fertilization regimes, compared with NoF, NPKM significantly increased the relative abundances of Planctomycetota, Verrucomicrobia, and Ascomycota, with an increase of 22.6, 15.1, and 13.3%, respectively. On the contrary, NPKM significantly increased the relative abundances of Chloroflexi, Gemmatimonadetes, Basidiomycota, Glomeromycota, and Chytridiomycota, with a decrease of 24.2, 13.1, 108.9, and 10.7%, respectively. For crop season, only the relative abundance of Bacteroidetes was 20.7% higher in the wheat fields than that in the rice fields. Compared with the lower soil layer, the relative abundances of Actinobacteria, Firmicutes, Alphaproteobacteria, Gammaproteobacteria, Rozellomycota, Mortierellomycota, and Chytridiomycota were significantly higher in the upper layer, while the relative abundances of Chloroflexi, Bacteroidetes, Nitrospirae, and Glomeromycota were significantly lower in the upper layer.

**TABLE 3:** Results of PERMANOVA and BETADISP testing for differences in multivariate dispersion between fertilization regimes, crop rotation, and soil depth in bacterial and fungal communities.

	Rice				Wheat			
	Bacteria		Fungi		Bacteria		Fungi	
	Pseudo-F	R^2^	Pseudo-F	R^2^	Pseudo-F	R^2^	Pseudo-F	R^2^
Soil depth	7.22***	0.247	2.60*	0.106	7.30***	0.25	3.58**	0.14
Fertilization regimes	3.54***	0.347	8.38***	0.557	2.22**	0.25	4.43***	0.4
**Pairwise fertilization regimes comparisons**								
	NoF (a)		NoF (a)		NoF (a)		NoF (a)	
	NPK (b)		NPK (b)		NPK (a)		NPK (a)	
	M (bc)		M (c)		M (ab)		M (b)	
	NPKM (c)		NPKM (d)		NPKM (b)		NPKM (b)	
**Multivariate homogeneity of groups dispersions**								
Fertilization regimes	8.25***		5.07*		1.1		3.11	

*Significant effects are indicated in bold. Different letters in the pairwise comparisons indicated significant difference at p < 0.05 (FDR-corrected). Results of BETADISP testing for differences in multivariate dispersion between fertilization regimes, crop rotation, and soil depth in bacterial and fungal communities.*

Regression of Bray-Curtis dissimilarity with soil variables revealed the influence of soil C and N on microbial community compositions (Table 4). For soil microbial communities in rice fields, the contents of soil TN, BRC, BRN, FRC, and FRN significantly correlated with bacterial and fungal community compositions, and SOC only affected soil fungal community compositions (*R*^2^ = 0.66, *p* < 0.001). In the wheat fields, FRC and FRN (bacteria *R*^2^ = 0.23, *p* < 0.05; fungi *R*^2^ = 0.26, *p* < 0.01) were significantly correlated with microbial community compositions, and fungi was also affected by SOC (*R*^2^ = 0.53, *p* < 0.001) and TN (*R*^2^ = 0.56, *p* < 0.001).

**TABLE 4:** Linear regression between soil microbial community composition and soil variables.

	Rice season				Wheat season			
	Bacteria		Fungi		Bacteria		Fungi	
	F	R^2^	F	R^2^	F	R^2^	F	R^2^
SOC	1.06	0.002	45.7	0.66***	3.83	0.11	27.2	0.53***
TN	4.62	0.14*	24.9	0.51***	2.46	0.06	30.6	0.56***
BRC	15.88	0.39***	5.51	0.16*	1.57	0.024	3.1	0.08
BRN	15.88	0.39***	5.51	0.16*	1.57	0.024	3.1	0.08
FRC	38.9	0.62***	10.91	0.3**	7.72	0.23*	9.07	0.26**
FRN	38.9	0.62***	10.92	0.3**	7.72	0.23*	9.07	0.26**

*Significance at p < 0.05, **significance at p < 0.01, ***significance at p < 0.001.

### Correlations Between Living and Dead Microorganism

**FIGURE 2:**
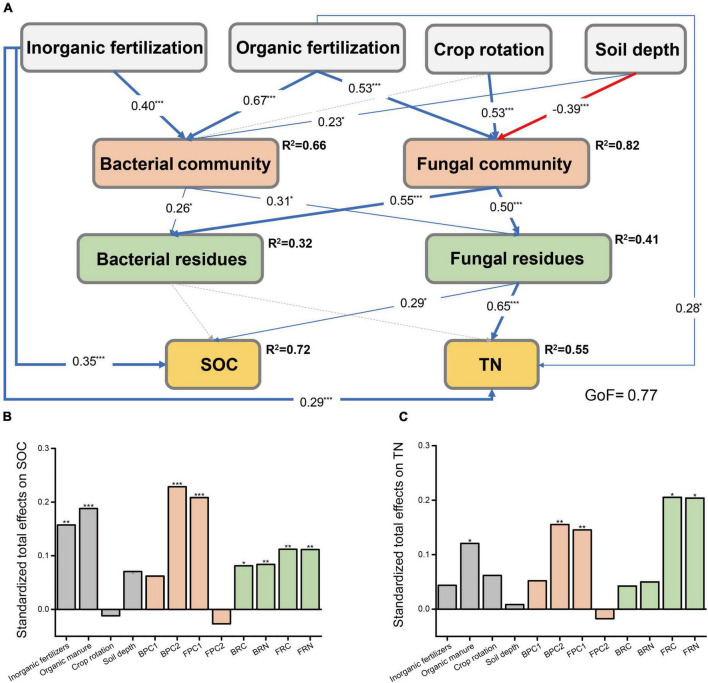
The partial least squares path models illustrating the effects of fertilization regimes, crop rotation, and soil depth on soil C and N by influencing the distribution of living and dead microorganism. **(A)** Bacterial and fungal communities were represented by the PC1 and PC2 of bacterial and fungal PCoA, respectively. Bacterial residues contained bacterial residues C and N. The blue and red lines indicated significant positive and negative correlations, respectively, while gray dash lines indicated insignificant correlation. The data on lines indicated the coefficient, and R^2^ indicated the coefficients of determination of the variables. GoF, goodness of fit. The cumulative bar plot displayed the standardized total effects of variables on SOC **(B)** and TN **(C)**. The asterisk indicated significant differences, *p < 0.05, **p < 0.01, ***p < 0.001. BPC1, sample values in PC1 axis of bacterial PCoA; BPC2, sample values in PC2 of bacterial PCoA; FPC1, values in PC1 of fungal PCoA; FPC2, values in PC2 of fungal PCoA; BRC, bacterial residues C; BRN, bacterial residues N; FRC, fungal residues C; FRN, fungal residues N.

Considering the significant correlations between microbial community compositions and microbial residues, we constructed a PLS-PM to access the direct and indirect effects of fertilization regimes, crop season, and soil depth on the contents of SOC and TN by affecting living and dead microorganisms in the soil (Figure 2). We found that inorganic fertilization increased soil SOC contents directly and indirectly by positively correlating with soil bacterial community compositions and increasing the contents of microbial residues C and N. Both organic fertilization and microbial communities exerted indirect positive impacts on SOC and TN contents. Relative to bacterial residues, fungal residues made more contributions to the contents of SOC and TN.

**FIGURE 3:**
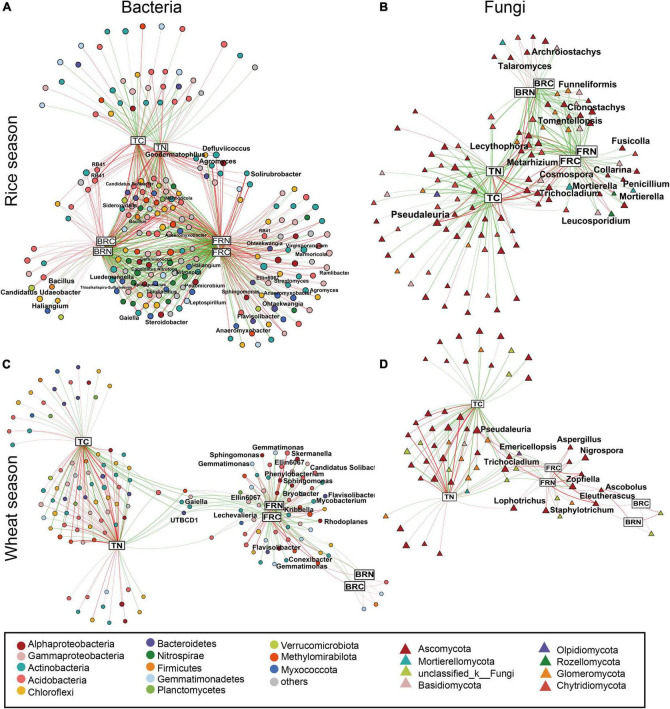
Association networks of microbial OTUs and soil variables. Bacterial networks in rice **(A)** and wheat **(C)** season, and fungal networks in rice **(B)** and wheat **(D)** season. OTUs are represented by circles, and soil variables by rectangle. Nodes’ sizes reflect their degrees, and are colored based on their taxonomic information. Green and red edges indicate positive and negative correlations, respectively. Wider edges reflect stronger correlations. BPC1, sample values in PC1 axis of bacterial PCoA; BPC2, sample values in PC2 of bacterial PCoA; FPC1, values in PC1 of fungal PCoA; FPC2, values in PC2 of fungal PCoA; BRC, bacterial residues C; BRN, bacterial residues N; FRC, fungal residues C; FRN, fungal residues N. Abbreviations: see Figure 2.

To further investigate the correlations between soil-specific microorganisms and microbial residues, we conducted a network analysis (Figure 3). Results revealed that both C and N in soil and microbial residues showed significant links to specific microbial OTUs. For the bacterial community in the rice fields, most OTUs affiliated to the phyla Actinobacteria, Acidobacteria, Chloroflexi, and Gammaproteobacteria showed significantly positive correlations with microbial residues, while the OTUs affiliated to the phyla Nitrospirae and Myxococcota showed significantly negative correlations with the microbial residues. On the contrary, in the wheat fields, there were fewer correlations between bacterial OTUs and bacterial residues. OTUs affiliated to Acidobacteria and Methylomirabilota were positively correlated with the fungal residues, while OTUs affiliated with Actinobacteria, Gammaproteobacteria, Alphaproteobacteria, and Gemmatimonadetes were negatively correlated. Considering their relative abundances, only Actinobacteria, which correlated positively with fungal residues, showed relatively higher abundance (1.85–6.42%, Supplementary Figure 1). Among all the identified bacterial genera, *Gaiella* (0.28–0.93%) and *Marmoricola* (0.17–0.86%) showed relatively higher abundances. Specifically, fertilization regimes and soil depth affected the genus *Gaiella*, which showed relatively higher abundances in the upper soil layer with M and NPKM treatments (Supplementary Figure 2). For the fungal community, most OTUs affiliated with the class Eurotiomycetes presented negative correlations with bacterial residues, and OTUs from the class Sordariomycetes showed negative links to the fungal residues, including the genera *Cosmospora*, *Nigrospora*, and *Zopfiella*. All these genera showed the lowest relative abundances in NPKM treatment than other fertilization treatments (Supplementary Figure 3). However, based on their relative abundances, only the class Pezizomycetes, which correlated positively with the fungal residues, presented a relatively higher abundance (0.22–61.09%), which was primarily attributed to the genus *Pseudaleuria*.

**FIGURE 4:**
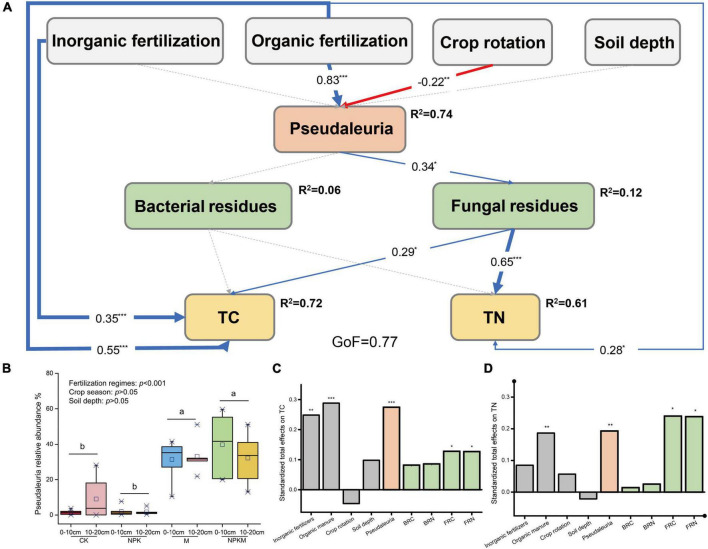
The partial least squares path models illustrating the effects of fertilization regimes, crop rotation, and soil depth on soil C and N by influencing the distribution of *Pseudaleuria* and microbial residues (for more details, see Figure 2). **(B)** The relative abundance of *Pseudaleuria* in different treatments and its difference between fertilization regimes, crop season and soil depth were test by LSD *t*-test. The different letters indicate significant difference. The standardized total effects of parameters on SOC **(C)** and TN **(D)**. Asterisks indicate significant differences. *p < 0.05, **p < 0.01, ***p < 0.001.

Compared with fertilization regimes, crop season, and soil depth, only fertilization regimes significantly affected the relative abundance of *Psedaleuria* (Figures 4B, *P* < 0.001), which showed higher abundances in M and NPKM with an average abundance of 34.1%. Considering its high relative abundance and significant correlations with fungal residues, we constructed the PLS-PM (Figure 4A) to further investigate the possible effects on microbial residues caused by *Psedaleuria*. Results revealed that the relative abundance of *Psedaleuria* was significantly positively correlated with fungal residues, which then indirectly increased the stocks of SOC and TN (Figures 4C,D, *p* < 0.01).

## Discussion

Both empirical studies and conceptual models have revealed that the incorporation of microbial biomass components into soils *via* microbial residues is large, which suggests that microbial inputs play a greater role in soil C sequestration (Lehmann and Kleber, 2015; Liang et al., 2019). Soil microorganisms convert plant C into microbial cell components and produce by-products in the process of decomposing plant residues (Liang et al., 2017). And compared with other cellular components, i.e., metabolites, microbial cell wall components are more stable due to their high polymerization (Gunina et al., 2017). After the death of microorganisms, the remnants of dead microbial cells serve as power for these biogeochemical processes because their chemical constituents persist as one of the largest C pools (Liang et al., 2017; Sokol et al., 2022). Soil bacteria and fungi are the dominant decomposers in soil, and their distinct physiologies are likely to differentially influence the rates of soil C cycling (Waring et al., 2013). In the present study, the long-term fertilization significantly stimulated the accumulations of soil bacterial residues, especially the application of organic manure, which was consistent with previous findings (Ding and Han, 2013; Faust et al., 2017). This was primarily because the addition of organic manure increased the nutrient resources and promoted the growth of soil bacteria, which preferred labile substrate (Schneider et al., 2010; Zhang et al., 2015). In addition, the microorganism carried in organic manure could also promote the accumulations of microbial residues (Ye et al., 2019; Chen et al., 2020).

The distinct microhabitats within the rice-wheat rotation also affected the compositions of microbial communities and their residues. In the present study, the contents of microbial residues in the wheat fields were much higher than the contents in the rice fields, as well as the contributions of microbial residues to soil C and N pools, especially the fungal residues. This is because the anaerobic environment in paddy soils inhibits the growth and metabolism of the fungal community (Moritz et al., 2009). Some studies have suggested that the high oxygen availability in the wheat fields enhances the turnover of microbial biomass, and increases the recolonization and decomposition of soil organic matter (Roth et al., 2011), which is inconsistent with our result. However, some studies demonstrated that high turnover rate of microbial biomass increased the incorporation of soil C and N into microbial residues, and higher temperatures in the rice season also enhanced the decomposition of SOC (Ma et al., 2018; Ding et al., 2019).

Microbial residues made a great contribution to soil C pools, and fungal residues contributed more than bacterial residues, which account for about 40% in most soils. The possible reason for that might be as follows. (1) Compared with bacteria, fungi have higher rates of growth and C assimilation, leading to a relatively high efficiency in utilizing C-containing substrates (Schneider et al., 2012). (2) Fungi are the main decomposer of cellulose, hemicellulose, and lignin, and can poly-condense the products into more stable C substrates (Roth et al., 2011; Schneider et al., 2012). (3) The amounts of the fungi hyphae are relatively huge, and their cell wall components are resistant to degradation. (4) Bacteria have relatively faster metabolic rates, and their residues are mostly readily degradable and easily utilized by microorganisms (Joergensen, 2018). Moreover, we found that crop season strongly affected the contribution of fungal resides to soil C and N pools, possibly due to the aerobic microhabitat in the wheat fields, which favored the fungal growth. Although no significant difference in the amounts of microbial residues was found between soil depths, higher ratios of microbial residues to SOC were observed in the upper soil layer, which indicated that soil microorganisms contributed more to soil C pools in surface soil layer.

The contents and components of microbial residues are also influenced by the soil microbial communities. A previous study has demonstrated that compared with other components of soil organic matter, soil microorganisms preferentially decompose and metabolize microbial residues, resulting in a rapid turnover of microbial residues (Zeglin and Myrold, 2013). As the soil-dominant microorganisms, bacteria and fungi contribute to the soil microbial residues that largely depend on their divergent responses to soil environment (Xia et al., 2019). Therefore, differences in the soil microbial communities directly affect the contents and compositions of soil microbial residues. In the present study, our results revealed that the long-term fertilization, especially the application of organic manure, favored the growth of soil microbial communities, and then increased the contents of microbial residues, leading to an enlarged soil C and N storage. And compared with bacterial residues, fungal residues contributed more to soil C and N pools. This is because the components in the bacterial cell walls, such as peptidoglycan, are easily degraded into labile C substrates, which are unfavorable to soil C sequestration (Joergensen, 2018). The SOC and TN were intrinsically coupled with the accumulations of microbial residues (Roth et al., 2011), which also supported our result.

Considering the strong correlations between microbial community compositions and their residues, we identified that some OTUs with relatively high abundances respond strongly to the accumulations of microbial residues. The much more complicated and significant correlations between FRC, FRN, and microbial OTUs indicated the stronger relationships between soil microbial communities and fungal residues. Among all microorganisms, the OTUs belonging to Actinobacteria showed the highest relative abundances, possibly due to the relative higher abundances of *Gaiella* and *Marmoricola*. However, their functions in mediating microbial residues were significantly weaker than fungal communities, especially the phyla Ascomycota, which contained a large proportion of saprotrophic fungi (Gourmelon et al., 2016). Previous studies have demonstrated that saprotrophic fungi are specialized in decomposing recalcitrant, ligno-cellulose containing materials, which involved in soil C cycling (De Boer et al., 2005; Lehmann and Rillig, 2015). Our study revealed that the fungal OTUs correlated with fungal residues were mostly classified into saprotrophic fungi, including *Zopfiella*, *Cosmospora*, *Nigrospora*, and *Pseudaleuria* (Tedersoo et al., 2014). Furthermore, the highest relative abundance of *Pseudaleuria* in all fungal OTUs was relatively higher in soils treated with organic manure, which is consistent with a previous study (Xiang et al., 2020). This was possibly because of the critical role of saprophytic fungi *Pseudaleuria* functioning in SOC cycling by secreting extracellular enzymes (Xu et al., 2012). Considering the strong and positive correlations with fungal residues and its relatively higher abundance, we suggested that the application of organic manure favored the growth of *Pseudaleuria*, then stimulated the accumulations of fungal residues, and finally benefited soil C sequestration.

## Conclusion

Microbial community compositions influence soil C sequestration by shaping the microbial set and by triggering changes in the concentrations and compositions of microbial residues. Our study found that the long-term fertilization, especially the application of organic manure combined with inorganic fertilizers, significantly increased the contents of C and N in soil and microbial residues. Compared with paddy soils in the rice season, upland soils in the wheat season accumulated more fungal residues by favoring the growth of fungi. Soil bacterial and fungal communities showed strong and complicated correlations with the contents of microbial residues, particularly the positive correlations between certain fungal taxa *Pseudaleuria* and fungal residues. These results suggested that the long-term application of organic manure significantly favored the growth of soil bacterial and fungal communities, and then stimulated the accumulations of microbial residues, particularly in fungal residues, leading to the enlargement of soil C and N pools. The quantitative impact of *Pseudaleuria* exerted on the contents and compositions of microbial residues needs further research.

## Data Availability Statement

The datasets presented in this study can be found in online repositories. The names of the repository/repositories and accession number(s) can be found in the article/Supplementary Material.

## Author Contributions

JC contributed to the sampling work and laboratory experiments, analyzed all the results of the experiments, and wrote the first draft of this manuscript. DS and HL contributed to the statistical analysis. DL helped to maintain the long-term experiment and support the sampling work. XW and JS revised this manuscript. WZ and GL guided and proofread the manuscript. All authors contributed to the article and approved the submitted version.

## Conflict of Interest

The authors declare that the research was conducted in the absence of any commercial or financial relationships that could be construed as a potential conflict of interest.

## Publisher’s Note

All claims expressed in this article are solely those of the authors and do not necessarily represent those of their affiliated organizations, or those of the publisher, the editors and the reviewers. Any product that may be evaluated in this article, or claim that may be made by its manufacturer, is not guaranteed or endorsed by the publisher.

## Footnotes

1
https://github.com/networkx/networkx

2
http://data.cma.cn/

3
https://ccb.jhu.edu/software/FLASH/index.shtml

4
https://github.com/OpenGene/fastp

5
https://submit.ncbi.nlm.nih.gov/subs/sra

6
https://github.com/vegandevs/vegan

7
https://matplotlib.org/

## Supplementary Material

Supplementary Figure 1 The relative abundance of microbial OTUs linked to microbial residues in the phylum/class level.

Supplementary Figure 2 The relative abundance of microbial OTUs linked to microbial residues in the genus level.

Supplementary Figure 3 The relative abundances of some genus under different fertilization regimes. Significance was test by ANOVA with LSD *t*-test.
