# Supplementary figures and images for: Living and Dead Microorganisms in Mediating Soil Carbon Stocks Under Long-Term Fertilization in a Rice-Wheat Rotation
Source: Front Microbiol. 2022 Jun 10;13:854216. doi: 10.3389/fmicb.2022.854216 (PMC9230992; doi:10.3389/fmicb.2022.854216)

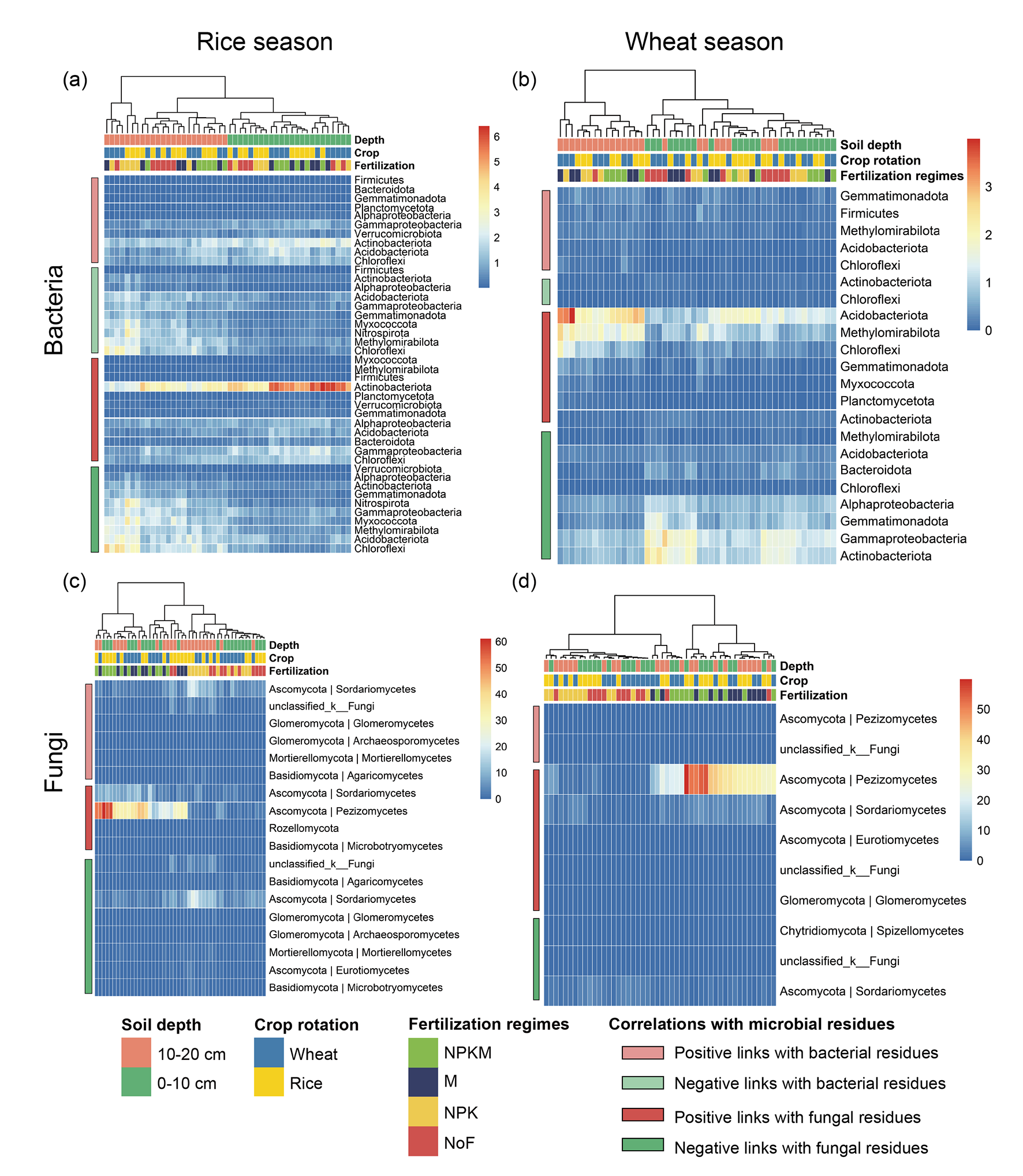

Supplement: Supplementary Figure 1 — The relative abundance of microbial OTUs linked to microbial residues in the phylum/class level. [file Image_1.tif]

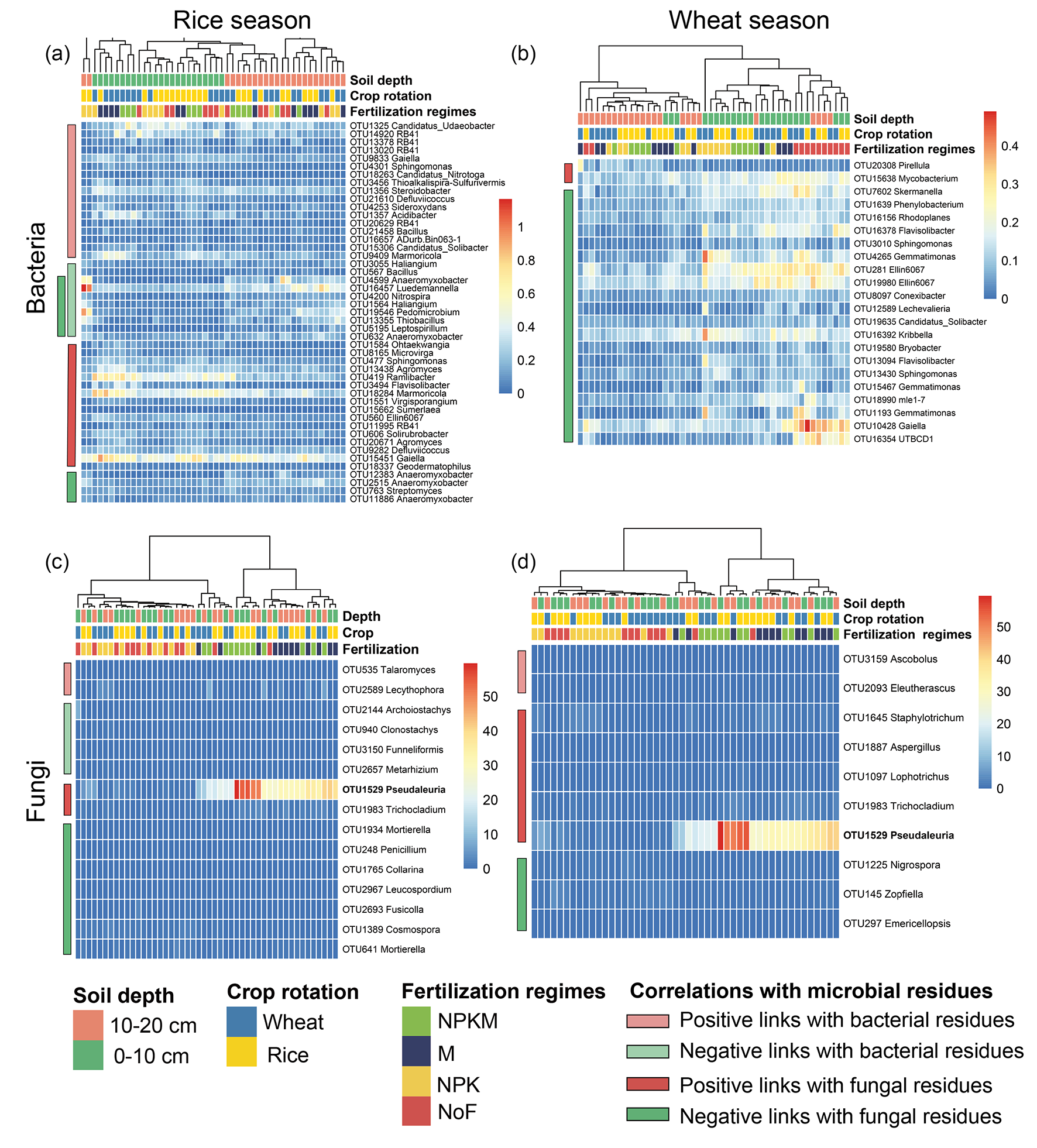

Supplement: Supplementary Figure 2 — The relative abundance of microbial OTUs linked to microbial residues in the genus level. [file Image_2.tif]

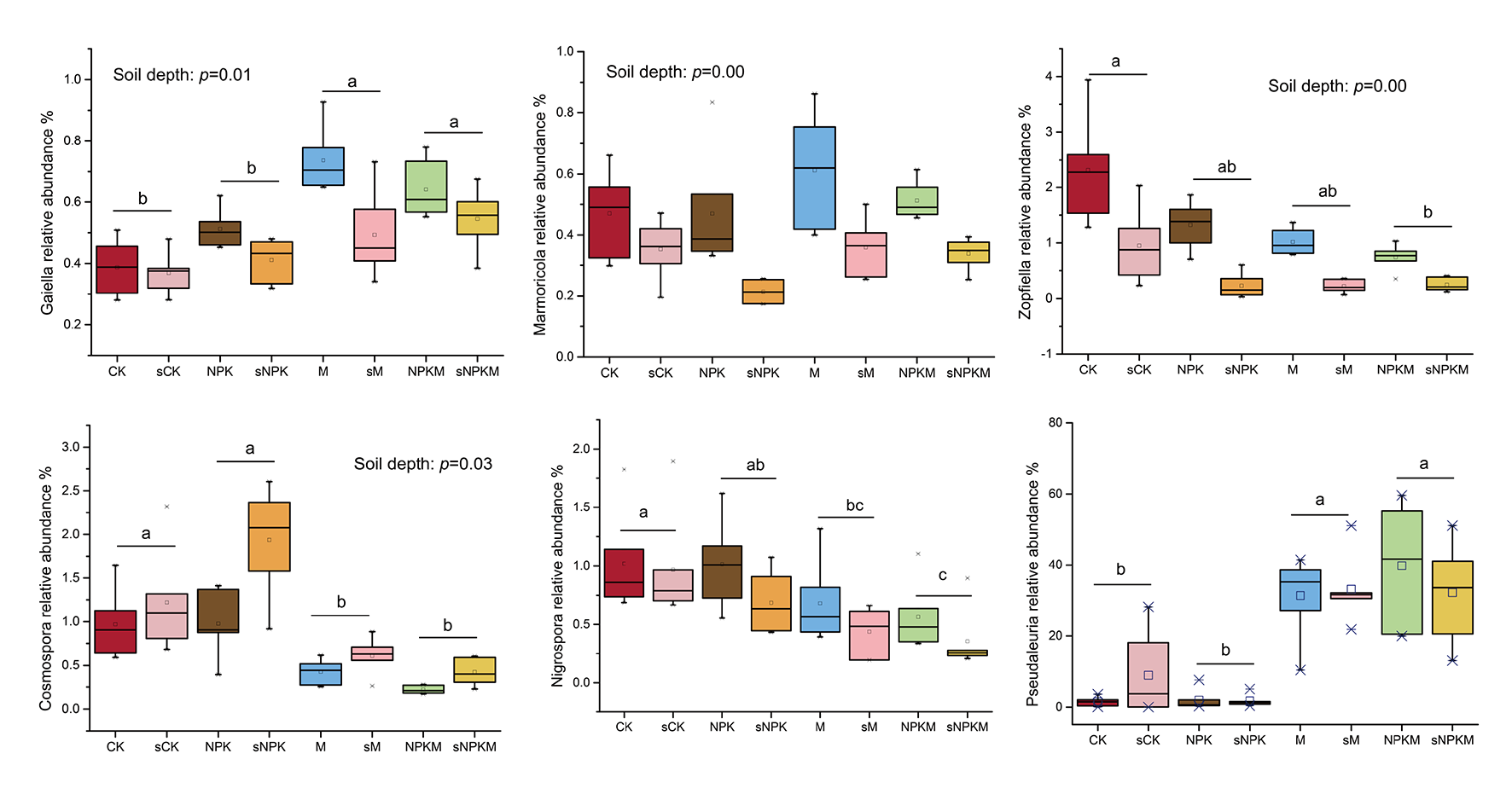

Supplement: Supplementary Figure 3 — The relative abundances of some genus under different fertilization regimes. Significance was test by ANOVA with LSD t-test. [file Image_3.tif]
